# Electroencephalographic Patterns in Chronic Pain: A Systematic Review of the Literature

**DOI:** 10.1371/journal.pone.0149085

**Published:** 2016-02-25

**Authors:** Eulália Silva dos Santos Pinheiro, Fernanda Costa de Queirós, Pedro Montoya, Cleber Luz Santos, Marion Alves do Nascimento, Clara Hikari Ito, Manuela Silva, David Barros Nunes Santos, Silvia Benevides, José Garcia Vivas Miranda, Katia Nunes Sá, Abrahão Fontes Baptista

**Affiliations:** 1 Graduate Program on Medicine and Health, School of Medicine, Federal University of Bahia, Salvador—BA, Brazil; 2 Laboratory of Functional Electrostimulation, Institute of Health Sciences, Federal University of Bahia, Salvador—BA, Brazil; 3 Research Institute on Health Sciences, University of Balearic Islands, Palma de Majorca, Spain; 4 Department of Speech-Language Therapy & Audiology, Institute of Health Sciences, Federal University of Bahia, Salvador—BA, Brazil; 5 Institute of Physics, Federal University of Bahia, Salvador—BA, Brazil; 6 Graduate and Research Program, Bahiana School of Medicine and Public Health, Salvador—BA, Brazil; Wadsworth Center, UNITED STATES

## Abstract

The main objective of this study is to review and summarize recent findings on electroencephalographic patterns in individuals with chronic pain. We also discuss recent advances in the use of quantitative Electroencephalography (qEEG) for the assessment of pathophysiology and biopsychosocial factors involved in its maintenance over time. Data collection took place from February 2014 to July 2015 in PubMed, SciELO and PEDro databases. Data from cross-sectional studies and longitudinal studies, as well as clinical trials involving chronic pain participants were incorporated into the final analysis. Our primary findings related to chronic pain were an increase of theta and alpha EEG power at rest, and a decrease in the amplitude of evoked potentials after sensory stimulation and cognitive tasks. This review suggests that qEEG could be considered as a simple and objective tool for the study of brain mechanisms involved in chronic pain, as well as for identifying the specific characteristics of chronic pain condition. In addition, results show that qEEG probably is a relevant outcome measure for assessing changes in therapeutic studies.

## Introduction

Chronic pain is caused by many conditions, but the etiology and the maintenance of pain symptoms over time still remains a scientific challenge. Although it seems obvious that the subjective nature of pain represents a relevant issue [1], recent evidence from neuroscience supports the idea that chronic pain can be understood not only as an altered perceptual state, but also as a consequence of several changes produced in neural processing after body injury or stress [2]. Recent experimental data have suggested that brain functioning and behavior might be different in individuals with chronic pain as compared to healthy ones [3,4]. Musculoskeletal injuries and the maintenance of chronic symptoms over time seem to affect both brain’s morphology and function [5].

Although there are several approaches for studying central mechanisms involved in chronic pain [6], quantitative Electroencephalography (qEEG) stands out as a valuable, non-invasive tool because it provides reliable and relevant information about brain functioning during rest, sensory stimulation and cognitive tasks [2]. In addition, this technique is safe, low-cost, and employs an easy methodology, thus making it an appropriate tool for use in clinical practice [7].

qEEG has been applied to assess brain functioning in several chronic pain syndromes [8–10]. Although many studies have shown that there are some common characteristics among individuals suffering from various pain syndromes, data remain inconclusive. In particular, two relevant questions will be addressed in this systematic review of qEEG studies in patients with chronic pain: (1) is there a characteristic pattern of EEG activity for chronic pain?, and (2) can EEG be used for diagnosis of chronic pain?

## Materials and Methods

### Search strategies and selection of studies

This review followed the guidelines of the Preferred Reporting Items for Systematic Reviews and Meta-Analyses–PRISMA [11] (available as Supporting Information, S1 Table). Data collection took place between February 2014 and July 2015 by searching in PubMed, SciELO and PEDro databases using the following criteria for eligibility: a) human population over the age of 18 years, with chronic pain of any origin, lasting for at least three months; b) observational studies with primary or secondary outcomes based on electroencephalographic data, or clinical trials with baseline qEEG data; c) publication date from January 2005 to July 2015. All studies examining qEEG parameters in humans were considered in the survey, including absolute and relative power, coherence, and degree of symmetry, evoked potentials (EP) and peak frequency of all bands.

The search descriptors in the database were “*qEEG and chronic pain* OR *qEEG and pain* OR *EEG and chronic pain* OR *EEG and pain* OR *coherence spectral and pain* OR *alpha power and pain* OR *theta power and pain* OR *beta power and pain* OR *delta power and pain* OR *somatosensory ERP or motor task and qEEG* OR *electroencephalography and pain”* and their equivalents in Portuguese and Spanish. Exclusion criteria included the following: studies involving experimentally induced pain; studies involving only healthy subjects or laboratory animals; acute pain and/or pain associated with neurological diseases such as stroke, schizophrenia, autism or brain tumors. The later studies were excluded in an attempt to eliminate confounders such as EEG changes due to psychiatric diseases or structural lesions of the central nervous system. EEG performed during sleep and other reviews were also excluded, as well as studies without control groups or those with less than four electrodes for EEG recording.

### Data extraction

Initially, two independent researchers (ESSP and DBNS) extracted data from the publication title and abstract. After reaching a consensus about selected studies based on the inclusion and exclusion criteria, full texts were retrieved for analyses. The following items were manually extracted, tabulated and described:

1. Study quality scored by using an adapted version of the Newcastle-Ottawa Scale [12–14] (Table 1);
2. Clinical and demographic characteristics, including number of participants per group, sex, age, diagnosis, diagnostic criteria, intensity and duration of pain (Table 2);
3. Study design, data collection and qEEG findings, including EEG protocols (amount and placement of electrodes, sampling rate), study merits and limitations (Table 3).

**Table 1:** Results of the quality assessment of studies and risk of bias using the Newcastle-Ottawa Scale.

Source	Criteria for patients selection	Use of any medication	Quality criteria from the Newcastle-Ottawa Scale			Total score (Up to 9 stars)
			Selection (Up to 4 stars)	Comparability between groups (Up to 2 stars)	Outcome (Up to 3 stars)	
Sarnthein *et al*., 2006	IASP^a^	Yes (antiepileptics, benzodiazepines, antidepressants and opiates)	3	1	2	6
Stern *et al*., 2006	IASP^a^	Yes (no neuroactive medication, benzodiazepines, antiepileptic drugs, tricyclic antidepressants, opioids—all doses informed on the original study)	3	1	2	6
Veldhuijzen *et al*., 2006	Study specific Inclusion/Exclusion criteria	Yes (paracetamol and/or NSAIDs)	3	2	2	7
Montoya *et al*., 2006	Study specific Inclusion/Exclusion criteria	Yes (antidepressants, analgesics/muscle relaxants/ non-steroidal anti-inflammatory drugs, anxiolytics)	3	2	2	7
Sitges *et al*., 2007	Study specific Inclusion/Exclusion criteria	Yes (antidepressants, analgesics/muscle relaxants/ non-steroidal anti-inflammatory drugs, anxiolytics)	4	2	2	8
Boord *et al*., 2008	Study specific Inclusion/Exclusion criteria	Yes Yes. (amitriptyline, diazepam, gabapentin, mexiletine, morphine, oxycodone, pregabalin, sodium valproate, tramadol and temazepam)	4	1	1	6
Bjork *et al*., 2009	Study specific Inclusion/Exclusion criteria	No	4	1	3	8
Sitges *et al*., 2010	Study specific Inclusion/Exclusion criteria	Yes (antidepressants, analgesics/muscle relaxants/ non-steroidal anti-inflammatory drugs, anxiolytics)	4	2	2	8
Bjork *et al*., 2011	Study specific Inclusion/Exclusion criteria	Same as above	4	2	3	9
Schmidt, *et al*. 2012	IASP^a^	Yes (antidepressants, analgesics/muscle relaxants/ non-steroidal anti-inflammatory drugs, anxiolytics)	4	2	2	8
Mendonça-de-Souza *et al*., 2012	Study specific Inclusion/Exclusion criteria	Not informed	3	0	1	4
Gonzalez-Roldan *et al*., 2013	Study specific Inclusion/Exclusion criteria	Yes (antidepressants, analgesics/relaxants/ non-steroidal anti-inflammatory drugs, anxiolytics)	3	0	2	5
De Vries, *et al*. 2013	Study specific Inclusion/Exclusion criteria	Yes (opioids, antiepileptics, benzodiazepines, antidepressants, lithium, non-steroidal anti-inflammatory drugs, paracetamol and proton pump inhibitors)	4	1	2	7
van den Broeke, *et al*. 2013	Study specific Inclusion/Exclusion criteria	No	4	2	2	8
Vuckovic, *et al*. 2014	Study specific Inclusion/Exclusion criteria	Yes (baclofen, carbamazepine, gabapentin, pregabalin, amitriptyline and diazepam)	3	0	2	5

a: International Association for the Study of Pain

**Table 2:** Study design, demographic and clinical characteristics of subjects from the included studies.

Source	Study design	Studied conditions (Diagnosis)	Diagnostic criteria	Patients with pain				Controls	
				N (w,m)^g^	Age in years Mean (SD) or Min—max	Pain intensity (0–10)	Pain duration in years Mean (SD)	N (w,m)^g^	Age in years Mean (SD) or Min—max
Sarnthein *et al*., 2008^1^	Clinical Trial	Neuropathic pain (different origins, severe forms)	IASP^a^	15 (6, 9)	62.1 (10.1)	6.9 (1.2)	Not informed	HC: 15 (8,7)	41–71
Stern *et al*., 2006	Clinical Trial	Neuropathic pain (different origins, severe forms)	IASP^a^	16 (7,9)	63 (10)	7.1 (1.4)	Not informed	HC: 16 (8,8)	56 (12)
Veldhuijzen *et al*., 2006^2^	Cross-sectional	Chronic pain of any origin	Not informed	14 (4,10)	47 (2.3)	6.8 (1.6)	7.9 (6.1)	HC: 30 (15,15)	48 (1.6)
Montoya *et al*., 2006	Cross-sectional	Fibromyalgia	ACRc^b^	15 (15,0)	49.7 (8.2)	7.3 (1.7)	13.5 (9.5)	HC: 15 (15,0)	48.0 (5.9)
Sitges *et al*., 2007	Cross-sectional	Fibromyalgia and Musculoskeletal pain (due to rheumatoid arthritis, radiculopathy, herniated disk)	Medical records and ACRc^b^	MSK: 18 (14,4) and FM: 18 (18, 0)	MSK: 46.4 (9.2) and FM: 49.4 (6.5)	MSK: 6.2 (1.8)and FM: 6.0 (2.1)	MSK: 6.4 (7.3)and FM: 11.7 (12.5)	HC: 16 (15,1)	49.2 (8.6)
Boord *et a*l., 2008	Cross-sectional	Neuropathic pain (secondary to paraplegia)	Medical records	8 (1,7)	35.3 (11.3)	Not informed	Not informed	PNP: 8 (0,8) and AB: 16 (1,15)	33.5 (10.3) 34.3 (10.7)
Bjork *et al*., 2009	Cross-sectional	Migraine with and without aura	Neurologist + IHSc^c^	33 (30, 3)	36.5 (12.7)	2.4 (.7) on a 0–4 scale	19.3 (11)	HC: 31 (28,3)	40.0 (11.4)
Sitges *et al*., 2010	Cross-sectional	Musculoskeletal pain (due to degenerative joint, intervertebrae disc, or inflammatory diseases)	Medical records	19 (19, 0)	48.4 (6.9)	5.2 (1.4)	6.2 (7.8)	HC: 21 (21.0)	40.5 (16.7)
Bjork *et al*., 2011^3^	Longitudinal	Migraine with and without aura	Neurologist + IHSc^c^	25 (23, 2)	34.7 (12.2)	2.6 (.5) on a 1–4 scale	19.0 (10)	HC: 18 (16,2)	38.5 (11.1)
Schmidt *et al*., 2012^2^	Cross-sectional	Low back pain	IASP^a^	37 (28,9)	50.0 (10.2)	Day of EEG: 4.5 (SD = NI) and month prior EEG: 5.7 (2.1)	13.4 (12.6)	HC: 37 (28,9)	49.8 (10.8)
Mendonça-de-Souza *et al*., 2012	Cross-sectional	Migraine with aura	IHSc^c^	11(11,0)	19–45	Not informed	Not informed	HC: 7 (7,0)	19–45
Gonzalez-Roldan *et al*., 2013	Cross-sectional	Fibromyalgia	ACRc^b^	20 (20,0)	53.4 (8.1)	6.0 (1.3)	18.3 (13.8)	HC: 20 (20,0)	52.7 (9.9)
De Vries *et al*., 2013	Cross-sectional	Chronic abdominal pain (due to chronic pancreatitis)	MCCS^d^	16 (6,10)	49.5 (11.9)	Severe^f^	5.4 (2.9)	HC: 16 (6,10)	48.0 (11.3)
van den Broeke *et al*., 2013^1^	Cross-sectional	Neuropathic pain (operated for unilateral breast cancer)	DN4^e^	8 (8,0)	52 54.3 (6.8)	Last 3 months:4.8 (1.4) and At the day: 1.9 (1.3)	Not informed	PNP: 11 (11,0)	53 (10.5)
Vuckovic *et al*., 2014^1^	Cross-sectional	Neuropathic pain (secondary to paraplegia)	Not informed	10 (3,7)	45.2 (9.1)	6.8 (1.6)	9.9 (6.3)	PNP: 10 (2,8) and AB: 10 (3, 7)	PNP: 44.4 (8.1) and AB: 39.1 (10.1)
			**Total**	**283**			**Total**	**287**	

SD = Standard deviation; PNP = Patients with no pain; AB = Able-bodied; HC = Healthy controls; NI = Not informed; FM = Fibromyalgia; MSK = Musculoskeletal.

^1^ Mean and SD of Age and Pain intensity computed by using data provided in the article

^2^ Mean and SD of Pain intensity and Disease duration computed by using data provided in the article

^3^ Combined means and SD computed by using data provided in the article.

^a^ International Association for the Study of Pain

^b^ American College of Rheumatology's criteria

^c^ International Headache Society's classification

^d^ Marseille and Cambridge Classification System

^e^ Douleur Neuropathique 4 questionnaire

^f^ Sample size, women / men

^g^ according to Marseille and Cambridge Classification System.

**Table 3:** Description of electroencephalographic (EEG) protocols, findings, strenghts and limitations of the included studies.

Source	Electrodes (total)	Sampling rate (Hz)	Pain condition	EEG Modality	Main outcomes	Results	Merit	Limitations
Sarnthein *et al*., 2006	60	250	Neuropathic pain	Spontaneous EEG at rest (closed eyes)	- Power spectral density (2–25 Hz) and average peak frequency	- 🡹 **Power delta, theta, alpha and beta (2–25 Hz) in patients with neurogenic pain** (nine were using centrally acting medication) compared to control group; - **🡻 average peak frequency (dominant peak shifted towards lower frequencies for the group of patients);** - **🡹 power delta, theta, alpha and beta (2–18 Hz)** for the subgroup of patients free from centrally acting medication, in the fronto-central electrodes.	- Well defined data preprocessing and editing;—follow up of seven patients 3 and 12 months after (central lateral thalamotomy).	- Nine (out of 15) patients used centrally acting medication
Stern *et al*., 2006	60	250	Neuropathic pain	Spontaneous EEG at rest (LORETA images, closed and open eyes conditions were pooled for analysis)	- Spectralpower density (alpha, beta, theta)	- **🡹 High theta power (6–9 Hz) activation in the pain matrix of neurogenic pain patients** (posterior insula, adjacent periinsular and inferior posterior parietal cortex) **and also 🡹 low theta power** (periinsular parieto-temporal cortex); - **🡹 low beta power activation in the pain matrix of neurogenic pain patients** (anterior cingulate, left dorsolateral prefrontal cortices and left insula) **and also 🡹 high beta power** (occipital lobe). S1, S3 and supplementary somatosensory cortices were also involved;—surgery decreased overactivation in the insula and insular cortices.	- Assessment before and after central lateral thalamotomy surgery to discuss an anatomo-physiological hypothesis;—well defined data preprocessing and editing.	- Nine (out of 16) patients used centrally acting medication.—small subgroup for after surgery follow up (6 out of 16)
Veldhuijzen *et al*., 2006	4	250	Chronic pain of any origin	EEG activity elicited by cognitive task	- Event related potential (P300 amplitude and latency)	- Controls showed **🡻** P300 amplitude at Oz for probe stimuli in a cognitive difficult task, when compared to an easy one. **This expected finding was not present in chronic pain patients**;—there was no difference between the groups regarding P300 latency.	Evaluation of data not usually studied by researchers.	Use of 4 electrodes
Montoya *et al*., 2006	32	1,000	Fibromyalgia	EEG activity elicited by somatosensory or auditory stimulation (two identical stimuli, S1 and S2,delivered with a 550 msec interval)	- Somatosensory event-related potentials	- **🡻** Event-related potentials amplitudes elicited by the somatosensory and auditory S2 (test stimulus) stimuli compared with those elicited by S1 (conditioning stimuli) stimuli in the healthy controls;—**fibromyalgia patients showed a lack of amplitude attenuation for somatosensory evoked potentials from S1 to S2, when compared to healthy controls.**	Clear and well defined EEG recording strategy.	- Did not explain clearly why only 9 of the 32 electrodes were analyzed. -did not analyze separately subjects taking antidepressants (> 50% of the sample).
Sitges *et al*., 2007	32	1,000	Fibromyalgia and musculoskeletal pain	EEG activity elicited by cognitive task	- Visual event-related potentials (P2)	- 🡹 **Visual event-related potentials (P2) amplitudes during exposure to affective pain descriptors, compared to pleasant words, in patients with chronic musculoskeletal pain;** - **🡹** visual event-related potentials (P2) amplitudes when looking at sensory pain descriptors than affective pain words in healthy controls, at parietal region.	Well defined data preprocessing and editing.	- Nearly 73% of patients with musculoskeletal pain and 60% of patients with fibromyalgia were taking centrally-acting medication (antidepressants).—no measure of illness or pain beliefs in chronic pain patients
Boord *et al*., 2008	14	2,048	Neuropathic pain	Spontaneous EEG at rest (closed and open eyes)	- Peak theta-alpha Frequency; Eyes closed/ Eyes open reactivity	- **🡻 Peak theta-alpha frequency in paraplegic patients with pain (closed eyes) compared to able-bodied and no-pain groups;** - **🡻** peak theta-apha frequency as an effect of medication in pain patients compared to both able-bodied and no-pain groups; - **🡻** EC/EO reactivity across all recording sites in pain group compared to both the able-bodied and no-pain groups.	Inclusion of medication as a factor in the analysis.	-Duration of pain is not specified;—Two (out of 8) patients used centrally acting medication
Bjork *et al*., 2009	12	256	Migraine with and without aura	Spontaneous EEG at rest (closed eyes)	- Absolute spectral power (delta, theta and alpha); Relative spectral power (band power/total power); Inter-hemispheric asymmetry	- **🡹 Relative theta power in migraineurs** in the parieto-occipital and temporal regions, when compared with controls;—the other relative power bands (alpha and delta), absolute power and asymmetry were similar between groups;—in age-adjusted analyses, headache intensity positively correlated with increased delta power and assymetry.	Detailed headache diaries completed before and after the tests.	- Selection of EEG segments for analysis was not clear—Results mostly representative of female migraineurs without aura
Sitges *et al*., 2010	32	1,000	Musculoskeletal pain	EEG activity elicited by somatosensory stimulation, while viewing pleasent and unpleasent images	- Somatosensory event-related potentials (P50); Power spectra; Entropy and fractal dimension of brain oscillations	- **🡻 Somatosensory event-related potentials on P50 amplitudes elicited by pleasant pictures in chronic musculoskeletal pain patients**, when compaed to healthy controls, at frontal (F3/F4), central (C3/C4) and temporal electrodes (T3/T4); - **🡻 theta and beta power in pain patients when viewing pleasant images**, when compared to healthy controls, over sensorimotor cortices and temporal regions; - **🡹** entropy in pain patients at P4 compared to P3 (parietal electrodes)–assymetry was not seen in healthy controls; - **🡹** values of fractal dimension at P4 than P3 (parietal electrodes) when pain patients were viewing unpleasant pictures. No significant differences due to hemisphere or affective condition were found on nonlinear measures for healthy controls;—no significant effects were observed in alpha1 (8–10 Hz) activity. In alpha2 (10–12 Hz) healthy controls had higher activity than chronic pain patients.	Well defined preprocessing and editing data.	- Nearly 72% of patients with chronic pain were taking centrally-acting medication (antidepressants)—Patients with chronic pain scored significantly higher than controls on measures of depression and anxiety
Bjork *et al*., 2011	21	256	Migraine with and without aura	EEG activity elicited by photostimulation (closed eyes)	- Steady state visual evoked EEG-responses (driving power)	- **🡻 Interictal photic driving in the beta range (18 Hz and 24 Hz) in migraineurs without aura, between attacks**; - **🡹 driving power in the alpha range (12 Hz) within 72 h before the attack**;—Attack trigger sensitivity, photophobia and pain intensity were negatively correlated with driving power asymmetry, while trigger sensitivity, photophobia and a family history of migraine negatively correlated with driving power.	Pain assessment between and after migraine attack (pain free).	- Selection of EEG segments for analysis was not clear—Different duration os stimulus
Schmidt *et al*., 2012	60	1,000	Low back pain	Spontaneous EEG at rest (closed eyes)	power indices: Grand-average power spectral density (peak power) and overall power; frequency indices: frequency of the dominant peak (peak frequency) and center of gravity	**- None of the four indices showed differences between low back pain participants and controls;**—correlations were found between EEG frequency (4–12 Hz) or power indices. Negative correlation between psychopathology symptoms, pain perception and frequency indices values. On the other hand, they observed positive correlation between frequency indices and life satisfaction. Finally, they also reported positive correlation among power indicies, pain ratings, and disease duration.	Current source density distribution algorithm was estimated.	- Eight outcome variables
Mendonça-de-Souza *et al*., 2012	6	200	Migraine with aura	Spontaneous EEG at rest (closed eyes) AND EEG activity elicited by photostimulation (closed eyes)	- Partial direct coherence among frontal, parietal and occipital regions	- **🡹 Partial directed coherence for migraine patients compared to controls in the baseline** (outside attacks) in parietal to frontal and frontal to occipital networks; - **🡻 coherence after non-nociceptive photic stimulation for migraine patients**, especially in the right frontal to parietal (F-P) and parietal to frontal (P-F) networks in both hemispheres (controls had an increase).	Authors used an understudied parameter in EEG analysis (partial coherence).	Selection of EEG segments for analysis was not clear
Gonzalez-Roldan *et al*., 2013	64	1,000	Fibromyalgia	EEG activity elicited by cognitive task (looking at happy, anger and pain faces)	- Evoked potential amplitude (N100) and spectral power	- **Patients had 🡹 theta power (all brain regions) in response to pain and anger faces than to happy faces for fibromyalgia patients. They also had 🡻 alpha power than pain-free controls to all faces in time window 250 to 500 ms after stimulus onset; - 🡹 N100 amplitudes to pain and anger faces in comparison with neutral faces for fibromyalgia patients, as compared to pain free controls**; - **🡹** N100 amplitudes for pain free controls to happy faces as compared to patients, and more positive event-related potential amplitudes to happy than to other faces in the time window of 200 to 300 ms;—No significant effects were found on alpha power between 250 and 500 ms or on beta power spectra (13–30 Hz).	Evoked Potential amplitude and wave amplitude parameters analysis in a cognitive context.	Fibromyalgia patients were more anxious and depressed than pain-free controls.
De Vries *et al*., 2013	26	500	Chronic abdominal pain	Spontaneous EEG at rest (closed eyes)	- Alpha power amplitude (spectral power) and peak Alpha frequency (center of gravity)	- **🡻 Peak alpha frequency (center of gravity) in chronic pancreatitis patients** compared with healthy controls (parietal and occipital regions);—No diferences in alpha power;—peak alpha frequency was negatively correlated with duration of the disease.	- Homogeneous group of patients with persistent visceral pain resulting from chronic pancreatitis.	- Nine (out of 16) patients used centrally acting medication.
van den Broeke *et al*., 2013	64	2,000	Neuropathic pain	Spontaneous EEG at rest (closed eyes)	- Alpha amplitude (spectral power) and alpha center of gravity	- **🡹 Alpha power (7.9–11.2 Hz) in patients with persistent pain after breast cancer** compared to patients without pain (no brain region specified);—the alpha frequency (center of gravity) was similar between patients with and without pain.	Additional analysis of the center of gravity frequency.	- Duration of pain was not reported, but it was not longer than one year;—six (out of eight) patients had pain intensity of 1 (on a 1–10 scale) at the experiment's day.
Vuckovic *et al*., 2014	61	250	Neuropathic pain	EEG at rest (closed and open eyes) and EEG activity elicited by cognitive task (imagery, open eyes)	- Power spectral densities (theta, alpha and beta);—Event-related desynchronization (ERD) and event-related synchronization (ERS)	- **🡻 Theta and alpha power spectral densities (or just power) in paraplegic patients with central neuropathic pain during imagination of movement of both nonpainful (arms) and painful limbs (legs)** compared to both healthy controls and paraplegics with no pain; - **🡹 theta and alpha power, in the relaxed eyes open state, for patients with pain (PWP)** when compared to patients with no pain (PNP).—the occipital alpha power in the eyes closed state was comparable between PWP and PNP groups; —PWP had higher ERD at Cz, spreading over all frequency bands during imagery (especially of the feet).**🡻**	Compared paraplegic patients with and without pain with able-bodied volunteers with no chronic pain.	Some patients in the group of paraplegic patients with pain were taken centrally acting medication.

### Quality assessment and risk of bias

Risk of bias was considered due to the heterogeneous diagnoses and symptoms. The analysis of exclusion criteria and patient selection, medication usage and validity of assessment instruments followed the following parameters:

1. Did inclusion and exclusion criteria follow the recommendations of the International Association for the Study of Pain (IASP) for diagnosis and classification of chronic pain, or did the study present detailed and consistent description of inclusion and exclusion criteria for patients and controls?
2. Were standardized assessment instruments used to determine the intensity and characterization of pain?
3. Did the study provide detailed information regarding type and dose of medication and temporarily avoided drugs that could alter the electroencephalographic recordings?

Study quality was quantified by using an adapted version of the Newcastle-Ottawa Scale [12], an instrument that assesses the quality of non-randomized studies included in systematic reviews and/or meta-analyses. The maximum scores of the modified scale were: four stars on the selection domain, two stars on the comparability domain and three stars on the outcome domain. The modified version used in this study (available as Supporting Information, S1 Fig) was based on previous adaptations of the original scale to cross-sectional studies/case-control studies [13,14]. Since the instrument does not have a cut-off score to categorize studies according to their quality, we considered only studies scoring above five stars as having moderate-to-good scientific quality [13]. It is not the goal of this study to analyze the clinical significance of EEG as a tool for detecting changes after clinical interventions.

## Results

### Selected studies

**Fig 1:**
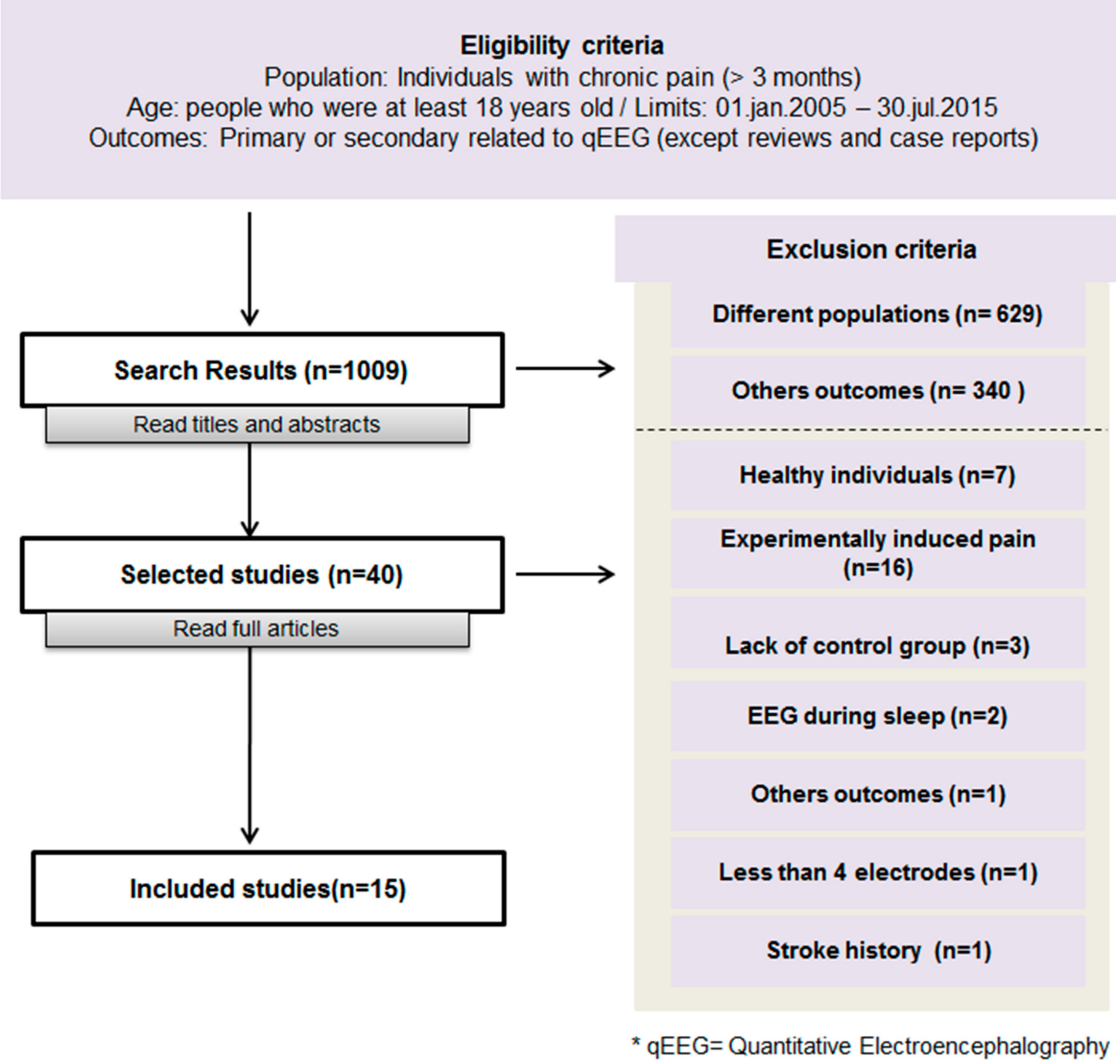
Flow chart of selection process for eligible studies.

According to the search criteria, we initially identified 1009 studies. Of these, 40 were selected for analysis after reading the abstract. We excluded another 25 due to the presence of the exclusion criteria, leaving a total of 15 studies which we selected for discussion [2,8–10,15–25] (Fig 1). Bibliographic information about the 40 articles selected for revision and the reasons for exclusion of the 25 studies are available in S2 and S3 Tables.

### Assessment of study quality and risk of bias

Table 1 displays the results of the quality assessment and risk of bias for the 15 studies included in this review. The quality scores on the Newcastle-Ottawa Scale ranged from four to nine stars. Detailed information about scoring for each study is provided on S4 Table. Classification of chronic pain established by the IASP was used as inclusion criteria in three studies. One study did not provide information about the status of the medication, while most of the studies described the drugs used by their patients and measures taken to control the biases resulting from this use. Two studies described the use of analgesics as exclusion criteria.

### Study design, demographic and clinical characteristics

#### Study design

The most frequently used type of design was cross sectional (n = 12), followed by clinical trials (n = 2) and longitudinal observational study (n = 1).

#### Patients’ profile

In total, 283 individuals with chronic pain and 287 controls were evaluated. The sample size of the patient groups ranged from eight to 37 individuals. Women were more frequently assessed than men (75.3% of all studies), and five studies included only females. The age of participants, when identifiable, ranged from 19 to 63 years.

The Visual Analogue Scale (VAS) was the most frequent instrument to measure the intensity of pain. The average pain intensity was above four (range from 0 to 10) in most studies (n = 10), and it was not reported in two studies. Eleven studies collected data about pain duration, which ranged between 5.2 and 19.3 years. Only four studies used instruments to characterize the pain. These results are presented at Table 2.

#### Diagnosis

The studies included participants with migraine (with and without aura), fibromyalgia, rheumatoid arthritis, osteoarthritis, low back pain, degenerative or inflammatory disc disease, neuropathic pain, radiculopathy, polyradiculopathy, polyneuritis, neuralgia, carpal tunnel syndrome, abdominal pain secondary to chronic pancreatitis, breast cancer and chronic pain of any origin. Most studies used standardized, validated and specific diagnostic criteria for chronic pain (n = 12) (Table 2).

#### EEG recording protocol

Technical protocols for EEG recording varied widely among studies. According to the authors, electrode placement followed the 10–20 International System in all studies, however the number of electrodes for EEG recordings varied from four to 64, with 10 studies (66.7%) reporting more than 25 electrodes. Sampling rates ranged from 200 to 2,048 Hz. EEG was recorded at least once during one of the following three experimental conditions: at rest, sensory stimulation (somatosensory, auditory or photic) and performance on cognitive tasks (Table 3). Some studies reported the use of more than one experimental condition for EEG recording.

#### qEEG findings

The most frequently analyzed parameters of brain activity were EEG power spectra at rest and the magnitude of different components of the event-related potentials (ERP) elicited by sensory stimulation or cognitive tasks. A brief description of the findings obtained at rest and in response to external stimulation is listed below. Nevertheless, it should be noted that the heterogeneity of the outcome variables prevented the standardization of data, making it impossible to conduct a meta-analysis.

Regarding spontaneous brain activity, eight studies collected spontaneous EEG data by asking participants to rest with either opened or closed eyes. The main EEG parameters analyzed in those studies were power spectra (or power density) [2,8,16,20,23,25] (n = 6) and peak frequency (n = 5) [2,9,10,20,23]. The analyses of power spectra revealed that chronic pain patients displayed increased theta (3.75–9 Hz) [8,16,20,25], alpha (7.75-13hz) [20,23,25], beta (12–30 Hz) [8,20], or delta band power (0,5–4 Hz) at rest [20].

Five studies were specifically designed to assess differences on spontaneous EEG by computing the dominant peak frequency from the average power spectra [2,9,10,20,23]. The major relevant finding from these studies was that the dominant peak of the power spectra shifted towards lower frequencies in chronic pain patients as compared with healthy controls.

Moreover, most studies on power spectra of spontaneous EEG activity have demonstrated that chronic pain patients displayed enhanced power spectra at frontal and parieto-occipital electrode locations. One study further compared the differences between chronic pain patients and controls on global spectral power by computing an estimation of the neural generators (source localization) within low theta (4–6 Hz), high theta (6–9 Hz), alpha (9–12 Hz), low beta (12–16 Hz), and high beta frequency band (16–30 Hz) [8]. The authors found that over-activations within high theta and low beta frequency bands were localized to multiple pain-associated areas, including insula, anterior cingulate, prefrontal, inferior posterior parietal, primary, secondary, and supplementary somatosensory cortices.

Some of the included studies assessed brain activity elicited by sensory stimulation or cognitive tasks. We included in this subsection all the studies using event-related potentials (ERPs) as potential markers of brain processing in chronic pain. Eight studies reported ERP changes when comparing patients with chronic pain and healthy controls [15,17–19,21,22,24,25]. Four studies reported significant reductions of several ERP components in response to different sensory and cognitive tasks [15,17,21,25]. These studies used ERPs to answer specific questions about sensory, affective and cognitive processing, making comparisons among them difficult.

The experimental tasks used in ERP studies included: photic stimulation during specific periods of migraine attacks [15,18]; auditory and somatosensory stimulation in individuals with fibromyalgia [19]; performance on cognitive tasks with varying difficulty in individuals suffering from different types of chronic pain [24]; reading pain descriptors in patients with fibromyalgia and musculoskeletal pain [22]; looking at faces expressing happy/anger/pain in individuals with fibromyalgia [17]; viewing pleasant/unpleasant/neutral pictures in patients with musculoskeletal pain [21]; and imagining painful/non-painful movements of limbs in participants with fibromyalgia [25].

The outcome variables used in these studies were also varied and included several parameters of the brain activity such as spectral [9,15,25]; amplitudes of early (P50) and late components (N100, P2, P300) of the ERPs (17,19,21,22,24]; and temporal dynamics of EEG signals—coherence [18], and non-linear parameters of brain activity such as fractal dimension and entropy [21].

Changes in EEG activity occurred in several brain areas and in more than one region in most studies. Changes elicited by photostimulation were found in O1 and O2 electrodes [15], as well as in P3, P4, and F4 electrodes [18]. Changes in brain activity elicited by cognitive were observed in almost all electrodes: Fz, Cz, Pz, and Oz [24]; T7, T8, P7, P8 [17], CZ [25], and F4, C4, CP4, TP8, P4, O2, F3, C3, CP3, TP7, P3, O1 [22]. Changes elicited by sensory stimulation were found at Fz, F3, F4, Cz, C3, C4, Pz, P3, P4 [19] and C3, C4, P3, P4, F3 F4 electrodes [21]. As changes of brain activity elicited by a stimulus or a task are very specific to each study, detailed information is provided in the description of each article in the next section (General overview of included papers).

Several of the studies computed correlations between behavioral or psychological features of pain and brain activity. For instance, significant positive correlations were found between pain intensity, delta power and asymmetry in migraine patients [16]. EEG power was negatively correlated with trigger sensitivity score, photophobia and family history of migraine [15]. In these patients, the asymmetrical distribution of enhanced power to photic stimulation was also negatively correlated with pain intensity. Disease duration was negatively correlated with the shift of the alpha dominant peak in chronic pancreatitis patients [2], but positively correlated with EEG power in back pain patients [10]. Frequency indices (frequency of the dominant peak and center of gravity) were negatively correlated with psychopathological symptoms and pain perception, and positively correlated with life satisfaction [10].

Table 3 describes all results for each study. S2 Table contains additional information, including a description of covariates.

#### General overview of included papers

A brief description of major findings reported by the selected studies is provided below. The first group of studies assessed spontaneous brain activity through EEG recordings.

Sarnthein *et al*. (2006) studied 15 patients with chronic neurogenic pain and 15 healthy controls (HC). Their experimental protocol included the evaluation of EEG power spectra at rest with opened (EO) and closed eyes (EC). They showed that patients had increased average power density in delta, theta, alpha, and beta ranges (from 2-25Hz). The peak frequency was shifted towards lower values in the presence of neurogenic pain. Discriminant analysis of peak frequency and peak height successfully identified patients, but it was not possible to use analysis of variance, due to non-parametric data distribution. These findings might also be related to the use of central acting drugs, like antiepileptics and benzodiazepines. Alpha peak was negative for HC in the parietal and occipital regions, and higher in the 13-15Hz range (high alpha/low beta) in frontal electrodes. Theta-beta coupling was abnormally high at fronto-parietal electrodes in patients, which was reversed by central lateral thalamotomy. This surgery successfully decreased pain in most of the studied patients (not all sample participants were submitted to surgery), decreasing theta power. However, it is possible that this result might have occurred because of the withdrawal of antiepileptic drugs. The authors proposed that the main findings on theta peak frequency and peak height was due to increased thalamocortical dysrhythmia.

Studying the same sample of neurogenic pain as Sarnthein *et al*. (2006), Stern *et al*. (2006) investigated 16 patients pre and post central lateral thalamotomy, compared to 16 HC. Data were analyzed by tomographic LORETA in the theta and beta ranges, in 15 regions of interest related to pain processing (pain matrix). They founda higher peak activation in the periinsular parieto-temporal cortex in low theta range (4-6Hz) for patients. The same occurred at low-beta range in the anterior cingular cortex, dorsolateral prefrontal cortex and left insula. Frequency dependent overactivation was shown in the 7-11Hz range (alpha-theta) for all regions, followed by a less intense peak in the lower-beta range (12-16Hz) in both right and left anterior cingulate, right dorsolateral prefrontal, mid cingulate cortices and posterior insula. Primary and secondary sensory cortices showed stronger activation in the left side. The use of central acting medications did not substantially change the results. Therapeutic central lateral thalamotomy lead to decreased overactivation of the right anterior cingulate cortex (8Hz–theta) and mid cingulate cortex (14Hz–beta).

Boord et al (2008) assessed participants with paraplegia, with (n = 8) and without (n = 8) neuropathic pain, comparing their resting EEG activity with healthy controls (n = 16). They found that peak theta-alpha frequency was shifted to lower frequencies at all recording sites in the presence of neuropathic pain, proposing that this phenomenon represented the presence of thalamocortical dysrhythmia. The use of medication was related to a decrease in theta-alpha frequency at three electrode locations (Pz, P3 and P7). They also analyzed eyes opened/eyes closed (EO/EC) ratio at all electrode locations in a broadband range (1-40Hz). Reactivity was decreased in the participants with neuropathic pain in 11 recording sites when compared to healthy controls, and in central (C3, C4) and frontal (F3, Fp1) electrodes, when pain participants were compared to participants without pain. These changes occurred in delta, theta, alpha and beta frequency ranges. The authors conclude that the slowing of EEG frequencies in neuropathic pain participants was attributed in part to the use of medications, but also as a consequence of pain. They also propose that EO/EC reactivity is a useful indicator of thalamocortical function, suggesting that there is a failed mechanism in the neurophysiological adjustment to sensory inputs in the neuropathic pain condition.

Bjork *et al*. (2009) compared interictal EEG recording from 33 migraineurs (6 with and 27 without aura) and 31 controls to estimate delta, theta, and alpha spectral power outside the period of crisis. They also evaluated absolute inter-hemispheric asymmetry. Data was collected at rest with closed eyes. Although they did not identify the number of electrodes utilized, they were grouped in three areas (parieto-occiptal, fronto-central and temporal). Differences between the groups were found only regarding increased theta power (all three regions), which was more pronounced in migraneurs without aura. Other secondary findings included a positive association between headache intensity, delta power, and delta asymmetry. An inverse correlation was also found between headache history, age and delta power/asymmetry. They argue that increased theta power may represent decreases in the frequency of thalamic activity, and delta findings represent latent cortical spreading depression.

Mendonça-de-Souza *et al*., (2012) assessed 11 women with migraine and visual aura, and seven HC. Their methods included EC resting EEG evaluation. They assessed partial coherence among occipital, parietal e frontal electrodes, within the frequency ranges of delta, theta, alpha, beta and gamma. Their results showed that in the basal period, migraneurs presented increased coherence in fronto-parietal and fronto-occipital networks. If the direction of the coherence was not considered, this phenomenon occurred in all frequency ranges, from delta to gamma.

Schmidt *et al*. (2012) assessed 37 low back pain participants and 37 HC to investigate the presence of thalamocortical dysrhythmia in the pain group. Their experimental protocol included 60 EEG electrodes recording at rest, with EO and EC. The clinical outcome variables were pain, quality of life and life satisfaction. EEG variables were average peak frequency, peak alpha power, peak alpha frequency, and overall power. They did not find significant differences in EEG indices between patients and controls, even after stratifying the patients into subgroups with and without neuropathic, or by pain severity. Correlations were only significant between EEG power and pain intensity and the presence of psychopathology. They conclude that thalamocortical dysrhythmia is not generally found in patients with moderate low back pain.

De Vries *et al*. (2013) compared the resting state EEG data from 16 patients with persistent abdominal pain due to chronic pancreatitis and 16 matched HC. They used 26 electrodes to acquire EEG data with EO and EC. Electrodes were grouped in central, parietal, occipital and frontal regions of interest and peak alpha frequency was estimated based on the center of gravity method. They did not find differences in alpha power between groups, but only in alpha peak frequency, which was skewed to lower frequencies in patients, predominantly in the parieto-occipital regions. Pain duration was inversely correlated with alpha peak frequency. They conclude that peak alpha frequency measures may represent a biomarker of chronic pain.

van den Broeke *et al*. (2013) studied two groups of patients after mastectomy, eight with pain and 11 without pain. They recorded EEG with 64 channels at rest and again, after a painful stimulation. This review only considered baseline data. Overall alpha EEG activity and alpha peak frequency (computed by the center of gravity method) were recorded only for parieto-occipital electrodes. Overall alpha amplitude, but not alpha peak frequency was higher in patients with pain. Neither alpha amplitude or frequency were correlated with pain intensity. However, pain intensity ranged from 3-6/10, which is considered to be low to moderate and is different from other studies where patients had severe neuropathic pain. The authors stated that their results are not sufficient to establish a clear role of alpha changes as a pain biomarker.

Vuckovic *et al*. (2014) evaluated two groups of 10 paraplegic patients, one with central neuopathic pain and another without. A third control group without any neurologic injury or pain was also enrolled. They recorded spontaneous EEG activity with 61 electrodes in EO state. During this state participants visually focused on a small cross. Paraplegics with pain showed larger theta power than those without pain, but were not different from controls. Patients with pain also presented greater alpha power than patients without pain. The dominant alpha frequency was lower in paraplegics with pain compared to those without pain. The authors suggest a specific EEG pattern for chronic pain.

A second group of studies assessed brain activity related to task performance elicited by sensory stimulation or cognitive tasks. They are described in the next paragraphs.

Montoya *et al*. (2006) examined brain habituation to repetitive, non-painful tactile and auditory stimulation in a sample of 15 female patients with fibromyalgia (FM) and 15 matched HC by using evoked-related potentials (ERP). Results indicate that amplitudes of early (P50) and middle somatosensory ERP components (160–360 ms) were reduced after repetition in HC, but not in FM patients. In addition, ERP amplitudes to auditory stimuli were reduced after repetition in both groups. The authors suggest the existence of an abnormal brain mechanism to specifically inhibit irrelevant somatosensory information in FM.

Veldzhijzen *et al*. (2006) studied 14 patients with either neuropathic or nociceptive chronic pain and 30 HC. They investigated whether resources are shared by pain and attention, and if allocation resources during pain are deficient. They studied ERP elicited by easy and difficult tasks associated with the recognition of pictures. Their results showed that HC presented smaller P300 amplitudes in Oz electrodes in the difficult task, and pain patients presented the reverse response. P100 amplitudes were larger in HC at Pz. The authors conclude that pain patients have abnormal attention allocation, but not attention capacity.

Sitges *et al*. (2007) examined the effects of negative mood on the brain processing of affective and sensory pain-related descriptors in 18 female patients with FM, 18 with chronic musculoskeletal (MSK) pain (rheumatoid arthritis, radiculopathy, herniated disk) and 16 female HC. Visual ERP elicited by pain descriptors and pleasant words were recorded while participants were making a decision about their appropriateness to define their pain experience (self-endorsement task). Results indicated that chronic pain patients reacted slower than HC participants, and that pain descriptors elicited higher positive ERP amplitudes than pleasant words in HC and MSK patients, with no differences in FM patients. These findings suggest an altered involvement of attentional and motivational brain systems in FM patients, as well as an excessive engagement of the motivational system during the processing of affective salient stimuli in patients with MSK pain.

Sitges *et al*. (2010) analyzed the brain dynamic of affective modulation of somatosensory processing in 19 female chronic pain patients (MSK) and 21 female HC. The experimental protocol included the recording of somatosensory ERPs elicited when participants were viewing affective pictures (pleasant, unpleasant and neutral). Results showed that pleasant pictures elicited an enhancement of the somatosensory P50 amplitudes in HC but not in patients with MSK pain. Moreover, power spectra of delta, alpha-2, beta-1 and beta-2 EEG frequencies were overall higher in HC compared to MSK patients. Finally, entropy and fractal dimension of EEG signal displayed a more asymmetrical distribution (right > left hemisphere) in MSK patients than in HC. These findings suggest that brain responses to somatosensory stimulation in chronic pain patients are abnormally modulated by the affective characteristics of the context in which body stimulation occurs. The reduction of alpha, beta and delta power, as well as the increased entropy over the right parietal lobe in MSK patients might indicate that persistent pain would lead to a sustained abnormal activation of the attentional brain system during the processing of non-painful body information.

Bjork *et al*. (2011) studied steady state visual evoked EEG-responses for 6, 12, 18 and 24 Hz flash stimuli in 33 migraineurs without aura, eight migraineurs with aura and 32 HC. EEG was recorded with 21 electrodes in an EC state. Intermittent photic stimulation was delivered for 10 seconds at each frequency, and repeated two times. Driving power (EEG power during photic stimulation) was analyzed and correlated with sensory hypersensitivity and severity of migraine. Driving responses to 18Hz and 24Hz were suppressed in the interictal phase in migraneurs without aura when compared to controls, probably representing a low-activation of the cerebral cortex. The same occurred at 18Hz when migraneurs were compared to controls, irrespective to the presence or not of aura. Additionally, the 18Hz driving power was more symmetric in migraneurs without aura when compared to those with aura or controls. Those participants with low threshold to photic stimulus also presented symmetric hemispheric activity, and were prone to present high sensitivity to light, sound, smell and physical activity. All these findings suggest a relationship between deficient habituation in migraneur patients.

Mendonça-de-Souza *et al*., (2012) also evaluated EEG inter-hemispheric coherence after 9Hz photic stimulation for 11 women with migraine and visual aura compared to seven HC. Photic stimulation was held through four rounds of 9Hz stimulation (3 seconds each, 0.2J, 20 cm in front of the eyes). EEG was recorded before, during and after stimulation, but only the latter was used for analysis. They showed that coherence decreases when photic stimulation starts in migraneurs, the opposite occurring in HC. When the stimulation ceased, migraneurs participants showed higher coherence than controls. They interpret these results as a resilient brain mechanism to deal with photic stimulation, a potential crisis triggering stimulus for migraine patients.

Gonzalez-Roldan *et al*. (2013) analyzed brain responses to viewing facial expressions of others’ pain in 20 female fibromyalgia patients (FM) and 20 HC by using visual ERPs. FM patients displayed higher N100 amplitudes to pain and anger faces as compared to neutral faces, as well as more reduced N100 amplitudes to happy faces in comparison with HC. By contrast, ERP amplitudes in the time-window 200–300 ms were more positive in response to happy than to the other faces in HC, but no differences were observable in FM patients. In addition, FM patients displayed higher theta power in response to pain and anger faces, as well as reduced alpha to all faces in comparison with HC. According to the authors, these findings suggest that FM patients could be characterized by an increased mobilization of early and preconscious attentional resources to biologically relevant cues during encoding processes and retrieval of sensory information

Vuckovic *et al*. (2014) also recorded EEG activity in EC and EO state (at rest) for three groups, including 10 paraplegic patients with central neuropathic pain, 10 paraplegic patients without central neuropathic pain, and 10 participants without any neurologic injury or pain. In the EC state, participants had to imagine hand or lower limb movements. There were no differences among the three groups regarding EEG power at this EC state. Paraplegic patients with or without pain showed no differences in alpha power in the EC state (imagery), compared to the EO state (rest), at posterior electrodes, which should increase in this state. This finding highlights a deficiency in EC/EO ratio. The dominant alpha frequency was lower in paraplegics with pain compared to those without pain. Event related desynchronization after imagination of movements was diffuse in the patients with pain, with no event related synchronization in the surrounding areas. The authors suggest this as a specific EEG pattern for chronic pain.

## Discussion

### Is there an EEG pattern for chronic pain?

The main objective of this review was to determine EEG patterns in the presence of chronic pain. Our findings show that there is a general trend towards increased power at lower EEG frequencies in patients with chronic pain at rest.

Increased theta power was observed in four out of six studies [8,16,20,25]. Among the included papers, increased theta EEG power at rest have been reported in patients with neuropathic pain [8,20,25], and migraine with or without aura [16], but not in low back pain patients, and fibromyalgia. It has been observed that neural sources of this increased theta power could be located in parts of the pain matrix such as prefrontal medial areas and anterior cingulate cortex [8]. Previous studies in laboratory animals have shown increased theta oscillations associated with thalamic dysfunction [26]. Moreover, it has been suggested that internally generated abnormal firing of thalamic neurons may disrupt thalamocortical networks and lead to abnormal pain processing [27–29]. This seems to be true in neuropathic pain conditions, where there is some degree of thalamic denervation, either bottom-up or top-down. A decreased inhibition of the thalamus seems to be linked to increased spikes of the neurons at around 4Hz, which would be the source of the increased theta power. This phenomenon has been called Thalamocortical Dysrhythmia (TCD) [27,28], a central nervous system dysfunction that can be associated with many painful disorders, and may be a marker of severe chronic neuropathic pain [29].

To confirm this hypothesis, Sarnthein *et al*. (2006) submitted patients with chronic neurogenic pain and increased theta power to central lateral thalamotomy, which successfully reversed this finding [20]. However, Schmidt *et al*. (2012) failed to identify TCD in low back pain patients, even when stratified for neuropathic pain due to root lesion, but this may have been due to inadequate statistical power or low pain severity [10]. Theta power increase was also found in migraine, a disease that shares many similarities with neuropathic pain, including central sensitization and central neuronal hyperexcitability [30]. Taken together, these results support the assumption that increased theta power may represent a biomarker of chronic severe neuropathic pain.

Alpha power at resting state was increased in patients with neuropathic pain [20,23,25]. Previous studies with cancer patients have shown similar results, which may be explained by the frequent cognitive dysfunction and fatigue found in those with this disease [31,32]. Other authors also found increased alpha in patients with neuropathic pain [33]. Wang *et al*. (2014) showed in patients with trigeminal neuralgia that thalamic metabolic alterations were correlated to cognitive dysfunction [34]. Alpha oscillations reflect inhibitory/excitatory mechanisms in the thalamocortical network [35], which are dysfunctional in neuropathic pain. However, data from experimental studies where pain was induced show that alpha power is not always increased, but conversely may be reduced in painful conditions [36–38]. We did not include these studies here, as we were looking for an EEG pattern for chronic pain, not acute pain. Also, alpha power suppression is associated with increased excitability of the sensorimotor cortex in ERP [39]. It is possible that acute crisis in chronic pain conditions may resemble experimental acute pain, which could explain alpha suppression. The same may occur in the presence of abnormal exacerbating pain behaviors, such as kinesiophobia and catastrophism [40], where excessive alertness may explain decreased alpha power [21,41,42].

We also observed that chronic pain was frequently associated with significant changes of early- and late-latency ERP amplitudes in response to somatosensory and visual pain-related information [15,21], suggesting an abnormal brain functioning linked to cognitive processes such as attention. Early-latency ERP components (including somatosensory P50 or visual N100) are basically related to early preconscious allocation of attentional resources to biologically relevant cues during encoding processes of sensory information which could be located on primary and secondary sensory brain areas [43]. By contrast, late-latency ERP components (P300, late positive amplitude) might reflect the activation of attention processes directed towards relevant stimuli that require rapid approach or avoidance responses and that are associated with the activation of a widespread brain network including the frontal cortex and the anterior cingulate cortex.

The analyses of ERP studies also reveal that the early allocation of attentional resources and the late engagement of motivational-based cognitive processes are altered in chronic pain patients during the processing of salient and bodily-relevant information. Thus, for instance, it has been shown that patients with fibromyalgia displayed an impaired short-term habituation to repetitive tactile stimulation, probably due to deficits in the coding and cognitive evaluation of somatosensory information arising from the body [19]. Moreover, an enhancement of N100 amplitudes to pain-related information is also a consistent finding in patients with chronic pain that has been linked to the establishment of tighter implicit memories for pain [17,41,44]. Finally, the fact that pain patients did not show a decrease in P300 amplitudes secondary to increasing difficulty of an attentional task with irrelevant stimuli, as observed in healthy controls [24], further demonstrates deficits in cognitive processes linked to pain processing. Consequently, it is possible that the stronger allocation of attentional resources and deficits in disengagement of attention observed in chronic pain patients during the processing of somatosensory and pain-related information might be a further consequence of neuroplastic changes due to central sensitization associated with chronic pain.

Pain modulates both cortical responses to external stimuli and internal events [17,19,45,46], and consequently is related to nociceptive events, but also to cognitive and psychological processes. Cognitive dysfunction is frequently observed in individuals with chronic pain [47,48], and correlated to decreased EEG responses to auditory [49] and visual [50] stimuli. As it is not commonly considered as a primary outcome measure in pain studies, cognitive dysfunction may represent a confounding factor, being the true source for diminished evoked potentials. However, this remains to be shown in studies assessing subgroups of patients according to their cognitive skills.

The reduction of ERP amplitudes may also be explained, in some situations, by the presence of habituation. Although sensitization is largely associated with the repetition of a stimulus or its maintenance in chronic pain, habituation to the repetitive unpleasant stimuli is also a pathophysiological response in this condition [6,51]. Cortical inhibition, which is dysfunctional in chronic pain, is associated to habituation, reinforcing the hypothesis that this phenomenon could explain the reduction of ERP amplitudes [52].

However, opposite results have been found in chronic pain patients [53,54]. In our review, fibromyalgia [19] was an example where habituation did not happen to repeated sensory stimulation, which may reflect that the pathophysiology of specific conditions may be a determinant factor for ERP responses. The nature of the event also seems to influence these brain responses. Thus, for instance, enhanced ERP amplitudes have been observed when unpleasant stimuli were presented: visual ERPs elicited by pain descriptors in patients with fibromyalgia and musculoskeletal pain [22]; visual ERP elicited by viewing pain and anger faces in patients with fibromyalgia [22]. These findings may indicate a significant alteration of brain processing in response to emotional stimuli in individuals with chronic pain [55,56].

Changes in EEG activity associated with chronic pain have been observed in different brain regions, including frontal, parietal, and occipital, or sensoriomotor and somatosensory regions. This widespread distribution of changes in brain processing is in agreement with findings provided by neuroimaging studies including functional Magnetic Resonance Imaging, Positron Emission Tomography, and Magnetoencephalography in patients with pain [39,40,57,58]. Accordingly, these findings support the idea that rather than a simple alteration of a specific 'pain center', there are multiple changes in an interconnected network of somatosensory, limbic and associative structures that receive inputs from multiple parallel nociceptive pathways [6].

Despite the heterogeneity in the type of pain and clinical characteristics of the participants included in the reviewed studies, there is agreement on the existence of diffuse abnormalities in sensory and motor information processing in patients with chronic pain. According to our review, lower ERP amplitudes and increased power of theta and alpha EEG oscillations seem to be the most consistent qEEG findings.

### Clinical applicability of qEEG in individuals with chronic pain

Self-report of pain is the principal outcome used by health professionals assessing patients with pain [1]. However, due to its subjective nature, self-report is not enough to provide information about the mechanisms involved in chronic pain, mainly because multiple intrinsic and extrinsic factors can influence the pain experience [59]. Therefore, the study of physiological markers that can reflect the underlying pain mechanisms is an important and relevant issue for clinicians. First, qEEG could help obtain an accurate diagnosis of pain based on objective parameters about the role of the central nervous system in the genesis and maintenance of pain. Second, qEEG could help improve the treatment of chronic pain by identifying patients who may benefit from therapies targeting central pain mechanisms [2].

Alterations of qEEG have been proposed as a biomarker in some distinct painful syndromes [6,17]. Electroencephalographic profiles of various populations with chronic pain are created with the rationale to identify the pathophysiology of pain [4] and promote the use of functional brain data as parameters of treatment success or failure [60]. The longer the exposure to pain, the greater are the changes in the alpha band of the EEG, indicating that this oscillation could be related to disease progression [2]. Changes in alpha also seem to predict the trend of throbbing pain in migraine [61] and disturbances in neural networks have been important to characterize acute pain [62]. Decreased beta synchronization after movement (SBPM) is present in pain syndromes of various origins [63–65], and seems to be related to increased cortical excitability. Its suppression could correspond to reduced inhibition of the motor cortex (disinhibition) [66]. However, our review did not identify changes in this bandwidth as the most frequent in individuals with chronic pain.

Interference of chronic pain on cognitive performance has also been examined by qEEG [67]. Data suggest that qEEG associated with sensoriomotor standardized protocols can help to improve the diagnosis of the mechanisms involved in the chronification process of pain over time [2,68].

### qEEG as a therapeutic biomarker in chronic pain conditions

The persistence of painful stimuli can generate maladaptive behavior by modifying the brain’s structure and function [3,69]. This condition is known as maladaptive plasticity [70]. Recent findings have repeatedly suggested that this phenomenon is fundamental for the chronification of pain symptoms [6]. Quantitative EEG may be used not only to identify some aspects of maladaptive plasticity, but it may also be a feasible, low cost alternative in the management of patients with chronic pain [68].

Imaging tools may help researchers and clinicians to assess the mechanisms and the efficacy of therapeutic interventions [71]. The recording of sensory and cognitive ERPs, for instance, has become a reliable biomarker for assessing the effects of various analgesic drugs [68,72,73]. Quantitative EEG can also document the inhibitory activity of the cortex in patients undergoing treatment with those therapies. Changes in alpha power and ERP amplitudes, have been described as outcome measures after Transcutaneous Electrical Nerve Stimulation (TENS) [74,75] and Kinesio Taping [76] studies. Alpha and theta peak frequencies have been recently used as markers for the therapeutic efficacy of Transcranial Direct Current Stimulation (tDCS) in individuals with neuropathic pain [77].

### Advantages and disadvantages of the EEG technique

In addition to providing data on brain electrical behavior in individuals with pain, EEG is a portable and low cost device compared to other techniques for the neurophysiological assessment of brain functioning. Subjects do not need to lay down, and there are no contraindications for the use of metallic implants in the body, enabling the evaluation of individuals with prosthetic devices.

Despite the advantages, qEEG faces some limitations: a) EEG data are extremely sensitive to external artifacts, including electromagnetic environmental factors; b) Data analysis is dependent on the theoretical knowledge of the evaluator; c) the technique has shown low accuracy in structural identification, in particular, deep brain structures [6].

Aside from the limitations inherent to EEG discriminating location power, technical and operational difficulties can be minimized:: 1) adequacy of the environment where the examination is performed. One should maintain an optimal temperature control, special lighting with gradual brightness adjustment or two options of brightness (strong and weak), quiet environment or a soundproof room and the shielding of instruments that may interfere magnetically on the record and 2) intensive team training on data acquisition and analysis.

### Limitations

Although our findings suggest that some patterns of brain activity of patients with chronic pain may exist, the review has limitations that must be taken into consideration. First and foremost, data from the included studies were very heterogeneous, which prevented a meta-analysis. Our conclusions are based on a qualitative analysis of the studies. Future studies should try to include similar variables, whenever possible, to allow for greater comparability of findings. Another limitation of this review was the exclusion of EEG sleep studies. We attempted to homogenize the sample, understanding that the awake standard EEG can be quite different from the sleep EEG. However, our findings may, in the future, be compared to findings of studies with sleeping participants in order to acquire a more comprehensive understanding of the chronic pain phenomenon.

Since we did not aim to analyze or discuss the clinical significance of EEG as a tool to detect changes after interventions, our findings and conclusions came from observational studies,. Clinical trials are considered the gold standard to provide the highest level of clinical evidence. However, our research questions are better addressed by the observational design. To control for quality of the evidence we presented here, the articles included were assessed by criteria defined by an adapted version of the Newcastle-Ottawa scale. In general, data acquisition, prossessing and analysis were clearly stated in these studies, which allow reproducibility of their methods.

## Conclusion and Perspectives

In conclusion, increased alpha and theta power at spontaneous EEG and low amplitudes of ERP during various stimuli seem to be clinical characteristics of individuals with chronic pain. Quantitative EEG can be a simple and objective tool for studying the mechanisms involved in chronic pain, identifying specific characteristics of chronic pain conditions and providing insights about appropriate therapeutic approaches. Nevertheless, more studies are necessary before drawing any conclusion on the utility of qEEG on chronic pain.

Further clinical studies should be conducted to establish the clinical applicability of this instrument as an effective marker for diagnosis and guide to strategies for pain control. Systematic reviews with samples of individuals who have similar characteristics and type of pain can help determine a specific EEG pattern for each type of chronic pain.

## Supporting Information

S1 Fig Newcastle-Ottawa Scale adapted for chronic pain and EEG findings cross-sectional studies. (PDF)

S1 Table PRISMA check list. (DOC)

S2 Table List of the 40 articles selected for analysis. (XLS)

S3 Table Characteristics of the 15 included studies. (XLS)

S4 Table Newcastle-Ottawa Scale scores of the 15 included studies. (DOC)
