# Supplementary material for: Electroencephalographic Patterns in Chronic Pain: A Systematic Review of the Literature
Source: PLoS One. 2016 Feb 25;11(2):e0149085. doi: 10.1371/journal.pone.0149085 (PMC4767709; doi:10.1371/journal.pone.0149085)
Supplement: S1 Fig — (PDF) [file pone.0149085.s001.pdf]

NEWCASTLE - OTTAWA QUALITY ASSESSMENT SCALE
ADAPTED FOR CROSS-SECTIONAL/ CASE-CONTROL STUDIES
Review: chronic pain and EEG patterns
Adapted from: Herzog et al., 2013[1] and; Eijkemans et al., 2012[2]

Note: A study can be awarded a maximum of one star for each numbered item within the Selection and Exposure categories. A maximum of two stars can be given for Comparability.

Selection (Maximum 4 stars)

1) Is the case definition (chronic pain) adequate?
   a) yes, with independent validation (eg. self reported doctor's diagnosis, reference to primary medical record source, international criteria) ❋
   b) yes, based on self reports
   c) no description

2) Representativeness of the cases
   a) Truly representative of the average in the target population. (all subjects or random sampling) ❋
   b) Somewhat representative of the average in the target population. (non-random sampling) ❋
   c) Selected group of users
   d) No description of the sampling strategy

3) Selection of Controls
   a) Matched by sex and/or age ❋
   b) Matched by other factor
   c) No description

4) Definition of Controls
   a) No history of pain ❋
   b) No description of source

Comparability (Maximum 2 stars)

1) Comparability of cases and controls on the basis of the design or analysis
   a) The study controls for the most important factor (anxiety/depression). ❋
   b) The study control for any additional factor. (eg. medication usage, duration of pain) ❋

Outcome (Maximum 3 stars)

1) Assessment of outcome (EEG findings)
   a) Independent blind assessment ❋
   b) Clear description of method for EEG data acquisition (number and placement of electrodes, equipment, sample rating) and data processing (how the parameters were extracted from the EEG data) ❋
   c) No description of method for EEG data acquisition and/or data processing

2) Statistical test
   a) The statistical test used to analyze the data is clearly described and appropriate, and the measurement of the association is presented, including confidence intervals and the probability level (p value). ❋
   b) The statistical test is not appropriate, not described or incomplete.

1- Herzog R, Álvarez-Pasquin MJ, Díaz C, Del Barrio JL, Estrada JM, Gil Á. Are healthcare workers' intentions to vaccinate related to their knowledge, beliefs and attitudes? A systematic review. BMC Public Health. 2013 Feb 19;13:154
2 - Eijkemans M1, Mommers M, Draaisma JM, Thijs C, Prins MH. Physical activity and asthma: a systematic review and meta-analysis. PLoS One. 2012;7(12):e50775.
