# Supplementary material for: Electroencephalographic Patterns in Chronic Pain: A Systematic Review of the Literature
Source: PLoS One. 2016 Feb 25;11(2):e0149085. doi: 10.1371/journal.pone.0149085 (PMC4767709; doi:10.1371/journal.pone.0149085)
Supplement: S4 Table — (DOC) [file pone.0149085.s005.doc]

| **Newcastle-Ottawa Scale item** | **Sarnthein et al., 2006** | **Stern et al., 2006** | **Veldhuijzen et al., 2006** | **Montoya et al., 2006** | **Sitges et al., 2007** | **Boord et al., 2008** | **Bjork et al. 2009** | **Sitges et al., 2010** | **Bjork et al., 2011** | **Schmidt, et al. 2012** | **Mendonça-de-Souza et al., 2012** | **Gonzalez-Roldan et al., 2013** | **De Vries, et al. 2013** | **Broeke, et al. 2013** | **Vuckovic, et al. 2014** |
| --- | --- | --- | --- | --- | --- | --- | --- | --- | --- | --- | --- | --- | --- | --- | --- |
| **A Selection (maximum 4)** | **3** | **3** | **3** | **3** | **4** | **4** | **4** | **4** | **4** | **4** | **3** | **3** | **4** | **4** | **3** |
| 1. Case definition adequate | 1 | 1 | 0 | 1 | 1 | 1 | 1 | 1 | 1 | 1 | 1 | 1 | 1 | 1 | 1 |
| 2. Representativeness of the cases | 0 | 0 | 1 | 0 | 1 | 1 | 1 | 1 | 1 | 1 | 0 | 0 | 1 | 1 | 0 |
| 3. Selection of controls | 1 | 1 | 1 | 1 | 1 | 1 | 1 | 1 | 1 | 1 | 1 | 1 | 1 | 1 | 1 |
| 4. Definition of controls | 1 | 1 | 1 | 1 | 1 | 1 | 1 | 1 | 1 | 1 | 1 | 1 | 1 | 1 | 1 |
|  |  |  |  |  |  |  |  |  |  |  |  |  |  |  |  |
| **B Comparability (maximum 2)** | **1** | **1** | **2** | **2** | **2** | **1** | **1** | **2** | **2** | **2** | **0** | **0** | **1** | **2** | **0** |
| 1. Controlled for most important factor | 0 | 0 | 1 | 1 | 1 | 0 | 0 | 1 | 1 | 1 | 0 | 0 | 0 | 1 | 0 |
| 2. Controlled for an additional factor | 1 | 1 | 1 | 1 | 1 | 1 | 1 | 1 | 1 | 1 | 0 | 0 | 1 | 1 | 0 |
|  |  |  |  |  |  |  |  |  |  |  |  |  |  |  |  |
| **C Outcome (maximum 3)** | **2** | **2** | **2** | **2** | **2** | **1** | **3** | **2** | **3** | **2** | **1** | **2** | **2** | **2** | **2** |
| 1. Independent blind assessment | 0 | 0 | 0 | 0 | 0 | 0 | 1 | 0 | 1 | 0 | 0 | 0 | 0 | 0 | 0 |
| 2. Description of methods | 1 | 1 | 1 | 1 | 1 | 0 | 1 | 1 | 1 | 1 | 1 | 1 | 1 | 1 | 1 |
| 3. Statistical test | 1 | 1 | 1 | 1 | 1 | 1 | 1 | 1 | 1 | 1 | 0 | 1 | 1 | 1 | 1 |
|  |  |  |  |  |  |  |  |  |  |  |  |  |  |  |  |
| **Total (maximum 9)** | **6** | **6** | **7** | **7** | **8** | **6** | **8** | **8** | **9** | **8** | **4** | **5** | **7** | **8** | **5** |

Table S1: Newcastle-Ottawa Scale scores of included studies.
